# Factors Associated with the Differences in Migraine Prevalence Rates between Spanish Regions

**DOI:** 10.1155/2014/323084

**Published:** 2014-01-30

**Authors:** Jorge Matias-Guiu, Cristina Fernandez, Jesús Porta-Etessam, Valentin Mateos, Samuel Diaz-Insa

**Affiliations:** ^1^Hospital Clínico San Carlos, 28010 Madrid, Spain; ^2^Service of Neurology, Institute of Neurosciences, Hospital Clínico San Carlos, Avenida Professor Martín Lagos S/N, 28010 Madrid, Spain; ^3^Medical Center of Asturias, 33191 Oviedo, Spain; ^4^Hospital Francesc de Borja, 46701 Gandía, Spain

## Abstract

We have analyzed the relation of prevalence rates in Spanish regions with a series of human, environmental, and ecological factors. We find that the variability in migraine rates found between Spanish regions may be explained by interregional differences in the percentage of daily smokers, percentage of alcohol consumers, percentage of population presenting physical and/or psychological life-limiting conditions, percentage of population engaging in physical exercise, minimum absolute temperatures per year, number of days under 0°C per year, and altitude.

## 1. Introduction

**Figure 1:**
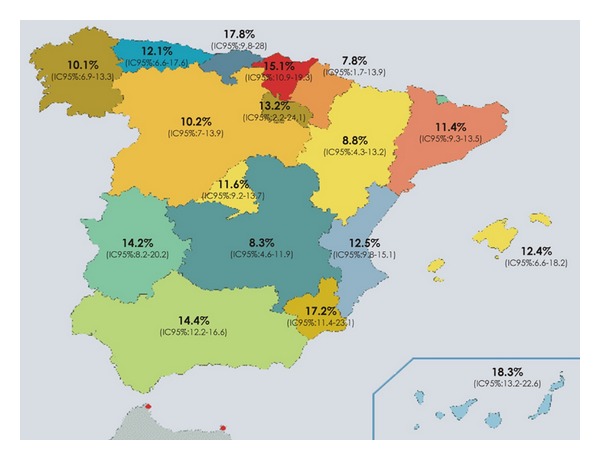
Regional migraine prevalence rates found in Spain (from 9). Galicia (10.1%), Asturias (12.1%), Cantabria (17.8%), Euskadi (15.1%), Navarra (7.8%), Catalonia (11.4%), Rioja (13.8%), Castilla-Leon (10.2%), Aragon (8.8%), Madrid (11.6%), Extremadura (14.2%), Castilla-La Mancha (8.3%), Valencia (12.5%), Baleares Islands (14.4%), Murcia (17.2%), Andalusia (14.4%), and Canary Islands (18.3%).

Differences in migraine prevalence rates have been described worldwide in different reports [1]; Asians have a lower prevalence than westerners [2, 3], and in Europe, they have been documented to be higher than in Africa [4] and of different values among countries [5] but not in all of studies [6]. Inside one country, there were significantly different regional prevalence rates in the United States [1], France [7], and Germany [8]. In Spain, our results have indicated that migraine prevalence rates vary between regions (Figure 1) ranging from 7.6% in Navarra to 18% in the Canary Islands [9].

Genetics, clinical conditions [10–12], environmental differences [13, 14], or lifestyle habits [15, 16] may participate in such variability [10–16]. Our aim was to identify factors that might explain the differences in migraine prevalence found between Spanish regions.

## 2. Methods

Regional one-year migraine prevalence rates were reported in a previous article [9]. Available data about demographic information, diseases, lifestyle habits, and environmental factors in every region of Spain were collected from the database of the National Statistics Institute of Spain, INE [17, 18]. This annual health survey was sampled between June 2006 and June 2007 from 31,300 households across 2,236 Spanish census districts. The number of households and census districts in each region was proportional to population of the region. The survey was mainly performed using personal interviews with subjects aged between 16 and 65 years and randomly selected from different households. Data about air pollutants were obtained from the Spanish register of emissions and pollutant sources [19]. Air pollutants emitted by a total of 6,368 registered facilities in Spain were quantified. The total emissions value for each region was calculated as the sum of the values of individual facilities located in the region. Weather data from each of the provinces in 2006 were taken from INE records [20]. Regional values were calculated as the median of the values for every province located in that region. Altitude data were obtained from the database pertaining to the National Geographic Institute [21]. The value for each region was calculated as the median altitude of the mountain ranges located in that region.

Excepting environmental factors, all other factors were given as the percentage of subjects in each region who meet a specific requirement. Lifestyle habits, clinical conditions, and physiological conditions comprised the human factors. Smoking habits, alcohol consumption, leisure-time physical activity, hours of sleep, and not having breakfast made up the series of lifestyle habits. Smoking habits were classified according to the smoking frequency, that is, daily smoker, occasional smoker (not daily), ex-smoker, or never-smoker. The number of cigarettes smoked by daily smokers was also taken into account. Alcohol consumption was given as the percentage of subjects having consumed alcohol in a specific period of time, that is, in the preceding two weeks or the preceding twelve months. Leisure-time physical activity was defined as the percentage of subjects habitually practicing any kind of sport or exercise during nonworking hours. Hours of sleep were calculated as the total time asleep reported by the subject, including naps. The following clinical and physiological conditions were taken into account: hearing loss (mild and severe), vision loss (mild and severe), hypertension, acute myocardial infarction, diabetes mellitus, hypo/hyperthyroidism, osteoarthritis, cervical back pain, chronic bronchitis, anaemia, duodenal ulcer, depression, hypercholesterolemia, obesity, and underweight. Obesity and low body weight were defined according to the body mass index (BMI), that is, underweight for a BMI < 18.5 and obese for a BMI ≥ 30.0 [22]. Life-limiting conditions and job stress were also included in the physiological factors due to their respective relationships with illness. Job stress was defined on a 7-point scale measuring work-related dissatisfaction and stress [23]. A life-limiting condition was considered to be any disease or health problem that may limit daily life activities of subjects. The life-limiting condition group was studied according to the degree of impairment, that is, negligible to severe, and the nature of the condition, that is, physical, psychological, or both. The study also considered reproductive variables, such as female fecundity rates by age, birth rates by population size, and mean number of children per woman. Environmental factors comprised air pollutants and weather conditions. Correlations with migraine prevalence were examined for the following air pollutants: carbon monoxide, carbon dioxide, nitrogen oxides, and sulphur oxides. Weather conditions examined in the study were number of days registering temperatures below 0°C or over 25°C in that year, total number of clear and cloudy days in the year, total annual rainfall (mm), maximum and minimum absolute temperature in the year, and altitude. Finally, inadequate indoor lighting, outdoor noises, neighbourhood disturbances, and neighbourhood pollution comprised the series of ecological factors. The response options for questions about ecological factors were 1 (no, not at all), 2 (yes, medium), or 3 (yes, high).

A nonparametric Spearman's rank correlation analysis was performed to evaluate the degree of association between differences in migraine prevalence and the series of explanatory factors. Spearman coefficient (*r*_*s*_) values range from +1 to −1, that is, from a perfect positive association to a perfect negative one. A zero value indicates no linear association between the variables. Furthermore, a simple linear regression was also performed for modelling the magnitude of the relationship between explanatory factors and the logarithm-transformed migraine prevalence values. We were used to establish the odds risk of the mean ratio that compares the mean of the prevalence rates among the regions when increasing the variable in a unit. Ratios were also estimated by exponentiating the beta coefficient values obtained from the simple linear regression. Independent explanatory factors presenting a significance *P* ≤ 0.05 or an association *r*_*s*_ ≥ 0.30 were analysed using a multiple linear regression model. The degree of fit of the data series with the regression model was indicated by the adjusted *R* ^2^ value. These values range from 1 to 0, that is, from perfect degree of fit to no fit. Those factors presenting statistical significance and/or clinical relevance composed the final multiple regression analysis. Colinearity between factors was also studied in every regression model. All the statistical procedures were performed using SPSS 15.0.

## 3. Results

The association analysis of migraine prevalence differences by a series of lifestyle habits is shown in Table 1. Percentages of daily smokers and alcohol consumers in last year were the only lifestyle habits to show a significant correlation (*P* = 0.04 and 0.03, resp.) with differences in migraine prevalence in the analysis of unhealthy habits. No significant correlation was found between migraines and the number of cigarettes smoked daily. While the correlation between migraine prevalence and daily smoking was positive (*r*_*s*_ = 0.49), the correlation with alcohol consumption was negative (*r*_*s*_ = −0.52). The linear regression beta coefficient was 0.04 (range 0.00–0.09) and −0.02 (range −0.04–0.00), respectively. Mean ratio values were 1.04 (range 1.00–1.09) and 0.98 (range 0.96–1.00). Factors such as leisure-time physical activity, sleep hours, and not having breakfast were also analysed as habits (Table 1). Of these listed factors, leisure-time physical activity showed a significant (*P* = 0.03) negative correlation (*r*_*s*_ = −0.53). The beta coefficient and mean ratio values were −0.02 (range −0.03–0.00) and 0.98 (range 0.97–1.00), respectively. The association analysis of the differences by a series of clinical and physiological conditions is shown in Table 2. No significant correlations were found between migraine prevalence and cervical back pain, chronic bronchitis, anaemia, duodenal ulcer, hypertension, acute myocardial infarction, or diabetes mellitus. Significant correlations were found with hypo/hyperthyroidism and osteoarthritis (*P* = 0.02 in both) with both correlations being negative (*r*_*s*_ = −0.57 and −0.56, resp.). The respective linear regression beta coefficient was −17.53 (range −35.37–0.30) and −3.02 (range −6.07–0.03). Mean ratio for both factors presented a value between 0 and 1. No significant correlations were found between migraine prevalence and body index factors, that is, obesity and low body weight. The combination of physical and psychological life-limiting conditions showed a significant (*P* = 0.04) negative correlation (*r*_s_ = −0.49) with migraine prevalence. The beta coefficient and odds ratio values were −2.85 (range −6.36–0.65) and 0.06 (range 0.00–1.92), respectively. None of the reproductive variables, which were female fecundity rates by age, birth rate by population size, and mean number of children per woman, presented significant correlations with prevalence (Table 3). No significant correlation was found between air pollutants and migraine prevalence. For weather conditions, minimum absolute temperature, total number of days with temperature below 0°C, and altitude showed a significant correlation (*P* = 0.01, *P* = 0.01, and *P* < 0.01, resp.). While minimum absolute temperature was positively correlated to migraine prevalence (*r*_*s*_ = 0.63), number of days with temperature below 0°C and altitude were negatively correlated (*r*_*s*_ = −0.63 and *r*_*s*_ = −0.70, resp.). The beta coefficient was 0.03 (range 0.01–0.06), −0.01 (range 0.01–0.00), and −0.06 (range −1.00–−0.02), respectively. Mean ratio values for the same variables were 1.04 (range 1.01–1.06), 0.99 (range 0.99–1.00), and 0.94 (range 0.91–0.98).

Before building a final regression model, the degree of significance of every group of factors was analysed by multiple linear regression. Models that significantly (*P* ≤ 0.05) explained the differences in migraine prevalence were lifestyle habits, life-limiting conditions, and weather conditions. Those factors presenting statistical significance and/or clinical relevance comprised the final multiple linear regression model (Table 4). A significant correlation was found for daily smokers (*P* = 0.05) and percentage of population presenting physical (*P* = 0.01) or psychological (*P* = 0.02) life-limiting conditions. The final regression model was statistically significant (*P* = 0.01) and it explained 61% of the variability in migraine prevalence.

## 4. Discussion

Our study suggests that variability might be explained by environmental factors and lifestyle habits. Our design provides a way to explore a wide range of population characteristics that might be associated with prevalence rates, but its main limitation was the lack of information about individuals so the rates were exposed to local confounding factors and that analyzed factors were limited to the information that was available in different national databases, but let us limit explanatory factors that could be tested in case-control studies.

Diabetes, hypertension, depression, and cardiovascular diseases are clinical conditions associated with headache prevalence [10–12], but none explain our data. Osteoarthritis or hypo/hyperthyroidism was negatively correlated but might be biassed by age. Association with stress has been documented [24] and in our results, prevalence of job-related stress was correlated with prevalence differences.

Environmental factors may be responsible for variability [25] that has been linked to cold temperature, high relative humidity, winds, precipitation, and cloudy days and in our study, the number of days with temperatures below 0°C and minimum absolute temperatures were significantly correlated. Although high altitudes have been associated with migraine [26], differences in altitude between regions were negatively correlated. None of the major air pollutants could be linked to migraine prevalence. Our study showed a positive correlation between the percentage of daily smokers and differences in prevalence in Spain. We found a negative correlation between drinking alcohol in the last year [15] and differences in physical exercise frequency [16].

## 5. Conclusions 

Sixty-one percent of variability of migraine prevalence between Spanish regions might be explained by interregional differences in percentage of daily smokers, percentage of alcohol consumers in a set period of time, percentage of population presenting physical and/or psychological life-limiting conditions, percentage of population engaging in physical exercise, minimum absolute temperatures per year, number of days under 0°C per year, and altitude.

## Figures and Tables

**Table 1:** Association analysis of the differences in migraine prevalence by the percentage of population in each region presenting a series of lifestyle habits.

	*r*_*s*_	*P* value	**β** (95% CI)	Mean R (95% CI)
Smoking habits				
Daily smokers	0.49	0.04	0.04 (0.00–0.09)	1.04 (1.00–1.09)
Occasional smokers	0.08	0.75	0.05 (0.11–0.21)	1.05 (1.12–1.23)
Ex-smokers	−0.41	0.10	−0.04 (−0.11–0.02)	0.96 (0.90–1.02)
Never-smokers	−0.20	0.44	−0.02 (−0.06–0.03)	0.98 (0.94–1.03)
Alcohol consumption				
In preceding 2 weeks	−0.40	0.11	−0.03 (−0.06–0.00)	0.97 (0.94–1.00)
In preceding 12 months	−0.52	0.03	−0.02 (−0.04–0.00)	0.98 (0.96–1.00)
Habits				
Leisure-time physical activity	−0.53	0.03	−0.02 (−0.03–0.00)	0.98 (0.97–1.00)
Hours of sleep	−0.04	0.87	−0.21 (−1.61–1.18)	0.81 (0.20–3.25)
Not having breakfast	0.11	0.66	0.02 (−0.12–0.15)	1.02 (0.89–1.17)

**Table 2:** Association analysis of the differences in migraine prevalence by the percentage of population in each region presenting a series of clinical and pathophysiological conditions.

	*r*_*s*_	*P* value	*β* (95% CI)	Mean *R* (95% CI)
Clinical conditions				
Mild hearing loss	−0.45	0.07	−4.88 (−10.78–1.01)	0.01 (0.00–2.75)
Severe hearing loss	−0.33	0.20	−18.64 (−67.11–29.83)	0.00 (0.00–9.02 × 10^12^)
Mild vision loss	0.10	0.69	5.30 (−6.27–16.88)	201.34 (0.00–2.13 × 10^7^)
Severe vision loss	0.15	0.57	11.71 (−32.43–55.85)	1.22 × 10^5^ (0.00–1.81 × 10^24^)
Hypo/hyperthyroidism	−0.57	0.02	−17.53 (−35.37–0.30)	0.00 (0.00–1.35)
Osteoarthritis	−0.56	0.02	−3.02 (−6.07–0.03)	0.05 (0.00–1.04)
Depression	−1.00	0.71	−0.84 (−4.55–2.86)	0.43 (0.01–17.53)
Hypercholesterolaemia	−0.31	0.22	−3.14 (−9.45–3.16)	0.04 (0.00–23.69)
Body mass				
Obesity	0.14	0.59	0.01 (−0.05–0.07)	1.01 (0.95–1.08)
Low body weight	−0.22	0.40	0.03 (−0.20–0.26)	1.03 (0.81–1.30)
Life-limiting conditions				
Severe impairment	0.40	0.11	2.26 (1.00–4.63)	9.63 (1.10–102.10)
Negligible impairment	−0.10	0.69	−1.17 (−3.01–0.67)	0.31 (0.05–1.96)
Physical limitation	0.31	0.22	1.43 (−1.41–4.27)	4.18 (0.24–71.52)
Psychological limitation	0.35	0.16	1.46 (−5.66–8.58)	4.31 (0.00–5.30 × 10^3^)
Both physical and psychological limitations	−0.49	0.04	−2.85 (−6.36–0.65)	0.06 (0.00–1.92)
Job stress	0.30	0.24	0.45 (−0.44–1.35)	1.58 (0.64–3.88)

**Table 3:** Association analysis of the differences in migraine prevalence by a series of environmental and ecological factors defining regions.

	*r*_*s*_	*P* value	*β* (95% CI)	Mean *R* (95% CI)
Air pollutants				
Carbon monoxide	−0.21	0.47	−0.21 (−1.86–1.44)	0.81 (0.16–4.21)
Carbon dioxide	−0.37	0.15	−1.66 (−4.11–0.79)	0.19 (0.02–2.20)
Nitrogen oxides	−0.16	0.53	−0.72 (−3.46–2.02)	0.49 (0.03–7.58)
Sulphur oxides	−0.34	0.20	−1.03 (−2.47–0.41)	0.36 (0.08–1.50)
Weather conditions				
Days registering *T*_*a*_ below 0°C	−0.63	0.01	−0.01 (−0.01–0.00)	0.99 (0.99–1.00)
Days registering *T*_*a*_ over 25°C	0.12	0.63	0.00 (0.00–0.00)	1.00 (1.00–1.00)
Clear days	−0.26	0.34	0.00 (−0.01–0.00)	1.00 (0.99–1.00)
Cloudy days	0.15	0.58	0.00 (0.00–0.01)	1.00 (1.00–1.01)
Total rainfall (mm)	0.03	0.91	0.00 (0.00–0.00)	1.00 (1.00–1.00)
Maximum absolute *T*_*a*_	0.13	0.63	0.01 (−0.04–0.06)	1.01 (0.96–1.06)
Minimum absolute *T*_*a*_	0.63	0.01	0.03 (0.01–0.06)	1.04 (1.01–1.06)
Altitude	−0.70	<0.01	−0.06 (−1.00–−0.02)	0.94 (0.91–0.98)
Ecological data				
Inadequate indoor lighting	0.05	0.84	0.38 (−3.34–4.10)	1.46 (0.04–60.64)
Outdoor noises	0.18	0.49	0.94 (−1.52–3.41)	2.57 (0.22–30.33)
Neighbourhood disturbances	0.15	0.56	0.87 (−1.09–2.84)	2.40 (0.34–17.06)
Neighbourhood pollution	0.25	0.34	1.24 (−1.50–3.98)	3.45 (0.22–53.57)

**Table 4:** Final multivariate linear regression model involving factors presenting statistical significance and/or clinical relevance.

	*P* value	**β** (95% CI)	Mean *R* (95% CI)
Daily smokers	0.05	0.03 (0.00–0.07)	1.04 (1.00–1.07)
Leisure-time physical activity	0.15	−0.01 (−0.03–0.00)	0.99 (0.97–1.00)
Physical life-limitation	0.01	3.82 (1.14–6.49)	45.51 (3.14–660.50)
Psychological life-limitation	0.02	7.98 (1.29–14.68)	2.92 × 10^3^ (3.62–2.36 × 10^6^)
Severe life-impairment	0.08	1.53 (−0.22–3.28)	4.63 (0.80–26.68)

	*P* value	Adjusted *R* ^2^	

Model significance	0.01	0.61	

## References

[1] Lipton RB, Scher AI, Kolodner K, Liberman J, Steiner TJ, Stewart WF (2002). Migraine in the United States: epidemiology and patterns of health care use. *Neurology*.

[2] Wong TW, Wong KS, Yu TS, Kay R (1995). Prevalence of migraine and other headaches in Hong Kong. *Neuroepidemiology*.

[3] Wang SJ (2003). Epidemiology of migraine and other types of headache in Asia. *Current Neurology and Neuroscience Reports*.

[4] Stovner LJ, Hagen K, Jensen R (2007). The global burden of headache: a documentation of headache prevalence and disability worldwide. *Cephalalgia*.

[5] Stovner LJ, Andree C (2010). Prevalence of headache in Europe: a review for the Eurolight project. *The Journal of Headache and Pain*.

[6] MacGregor EA, Brandes J, Eikermann A (2003). Migraine prevalence and treatment patterns: the global Migraine And Zolmitriptan Evaluation survey. *Headache*.

[7] Lantéri-Minet M, Valade D, Géraud G, Chautard MH, Lucas C (2005). Migraine and probable migraine—results of FRAMIG 3, a French nationwide survey carried out according to the 2004 IHS classification. *Cephalalgia*.

[8] Pfaffenrath V, Fendrich K, Vennemann M (2009). Regional variations in the prevalence of migraine and tension-type headache applying the new IHS criteria: the German DMKG Headache Study. *Cephalalgia*.

[9] Matías-Guiu J, Porta-Etessam J, Mateos V (2011). One-year prevalence of migraine in Spain: a nationwide population-based survey. *Cephalalgia*.

[10] Kurth T, Gaziano JM, Cook NR (2007). Migraine and risk of cardiovascular disease in men. *Archives of Internal Medicine*.

[11] Kurth T, Schürks M, Logroscino G, Gaziano JM, Buring JE (2008). Migraine, vascular risk, and cardiovascular events in women: prospective cohort study. *British Medical Journal*.

[12] Patel NV, Bigal ME, Kolodner KB, Leotta C, Lafata JE, Lipton RB (2004). Prevalence and impact of migraine and probable migraine in a health plan. *Neurology*.

[13] Hoffmann J, Lo H, Neeb L, Martus P, Reuter U (2011). Weather sensitivity in migraineurs. *Journal of Neurology*.

[14] Zebenholzer K, Rudel E, Frantal S (2011). Migraine and weather: a prospective diary-based analysis. *Cephalalgia*.

[15] Aamodt AH, Stovner LJ, Hagen K, Bråthen G, Zwart J (2006). Headache prevalence related to smoking and alcohol use. The Head-HUNT Study. *European Journal of Neurology*.

[16] Varkey E, Hagen K, Zwart JA, Linde M (2008). Physical activity and headache: results from the Nord-Trøndelag Health Study (HUNT). *Cephalalgia*.

[17] Instituto Nacional de Estadística INEbase: National Health Survey. http://www.ine.es/jaxi/menu.do?type=pcaxis&path=/t15/p419&file=inebase&L=1.

[18] Instituto Nacional de Estadística INEbase: Municipal Register. http://www.ine.es/jaxi/menu.do?type=pcaxis&path=/t20/e260&file=inebase&L=1.

[19] Registro Estatal de Emisiones y Fuentes Contaminantes Public Information: pollutants releases. http://www.en.prtr-es.es/informes/pollutant.aspx.

[20] Instituto Nacional de Estadística INEbase: Climatology. http://www.ine.es/jaxi/menu.do?type=pcaxis&path=/t43/a012/a1998&file=pcaxis.

[21] Instituto Geográfico Nacional National atlas of Spain: altitude. http://www.ign.es/ign/layoutIn/anetabladatosdatosgeneralesgeneral.do?tipoBusqueda=altitudes.

[22] Prentice AM, Jebb SA (2001). Beyond body mass index. *Obesity Reviews*.

[23] Matteson MT, Ivancevich JM (1982). Stress and the medical technologist. I: a general overview. *The American Journal of Medical Technology*.

[24] Wöber C, Holzhammer J, Zeitlhofer J, Wessely P, Wöber-Bingöl Ç (2006). Trigger factors of migraine and tension-typeheadache: experience and knowledge of the patients. *Journal of Headache and Pain*.

[25] Friedman DI, de ver Dye T (2009). Migraine and the environment. *Headache*.

[26] Wilson MH, Newman S, Imray CH (2009). The cerebral effects of ascent to high altitudes. *The Lancet Neurology*.

